# Variability in chemotherapy delivery for elderly women with advanced stage ovarian cancer and its impact on survival

**DOI:** 10.1038/sj.bjc.6604298

**Published:** 2008-03-18

**Authors:** J D Wright, T Doan, R McBride, J S Jacobson, D L Hershman

**Affiliations:** 1 Department of Obstetrics and Gynecology, Herbert Irving Comprehensive Cancer Center, College of Physicians and Surgeons, New York, NY 10032, USA; 2 Department of Epidemiology, Mailman School of Public Health, Columbia University, New York, NY 10032, USA; 3 Department of Medicine, Herbert Irving Comprehensive Cancer Center, College of Physicians and Surgeons, New York, NY 10032, USA; 4 New York Presbyterian Hospital, New York, NY 10032, USA

**Keywords:** ovarian cancer, chemotherapy, treatment delay, duration of chemotherapy

## Abstract

Given the survival benefits of adjuvant chemotherapy for advanced ovarian cancer (OC), we examined the associations of survival with the time interval from debulking surgery to initiation of chemotherapy and with the duration of chemotherapy. Among patients ⩾65 years with stages III/IV OC diagnosed between 1991 and 2002 in the Surveillance, Epidemiology, and End Results-Medicare database, we developed regression models of predictors of the time interval from surgery to initiation of chemotherapy and of the total duration of chemotherapy. Survival was examined with Cox proportional hazards models. Among 2558 patients, 1712 (67%) initiated chemotherapy within 6 weeks of debulking surgery, while 846 (33%) began treatment >6 weeks. Older age, black race, being unmarried, and increased comorbidities were associated with delayed initiation of chemotherapy. Delay of chemotherapy was associated with an increase in mortality (hazard ratio (HR)=1.11; 95% CI, 1.0–1.2). Among 1932 patients in the duration of treatment analysis, the 1218 (63%) treated for 3–7 months had better survival than the 714 (37%) treated for ⩽3 months (HR=0.84; 95% CI, 0.75–0.94). This analysis represents one of the few studies describing treatment delivery and outcome in women with advanced OC. Delayed initiation and early discontinuation of chemotherapy were common and associated with increased mortality.

Over the past three decades, advances in chemotherapy, including platinum- and taxane-based regimens and intraperitoneal chemotherapy, have improved survival among women with advanced ovarian cancer (OC) (Alberts *et al*, 1996; Markman *et al*, 2001; Armstrong *et al*, 2006).

Failure to receive standard therapy is associated with decreased survival (Tingulstad *et al*, 2003). However, not all patients, especially elderly patients, are offered optimal chemotherapy. A study by our group found that although 83% of elderly patients with stage III and IV disease received some form of chemotherapy, only 52% received platinum-based therapy. Factors associated with non-platinum-based treatment included older age, nonwhite ethnicity and number of comorbid medical conditions (Sundararajan *et al*, 2002). Provider type and surgical volume have also been shown to influence the likelihood of receiving chemotherapy (Schrag *et al*, 2006).

Dose-intense treatment (higher mg per m^2^ per unit of time) has been found to improve the survival of patients with breast cancer and other cancers (Hudis *et al*, 1999; Citron *et al*, 2003). But most clinical trials among patients with OC have failed to show a survival advantage of dose-intense regimens (McGuire *et al*, 1995, 1996; Conte *et al*, 1996; Jakobsen *et al*, 1997; McGuire, 1997; Gore *et al*, 1998). For example, the Gynecologic Oncology Group (GOG) compared patients treated with eight cycles of standard dose cisplatin and cyclophosphamide to those receiving four cycles of high-dose therapy. Although response rates and survival were similar, toxicity was more common in the dose-intense group than in the standard dose group (McGuire *et al*, 1995).

The optimal timing of chemotherapy initiation in the postoperative period and the ideal duration of treatment (including number of cycles) have not been determined. However, in most clinical trials, patients are required to register within 6 weeks of surgery. Studies examining the time from surgery to initiation of adjuvant chemotherapy have reported varied results; however, some have suggested that prolonging the interval between surgery and the initiation of adjuvant chemotherapy adversely affects survival (Omura *et al*, 1989; Warwick *et al*, 1995; Flynn *et al*, 2002; Gadducci *et al*, 2005; Paulsen *et al*, 2006a; Aletti *et al*, 2007).

The goal of our study was to analyse the association of demographic and clinical factors with delay in initiation of chemotherapy and early termination of chemotherapy in women with advanced OC and to evaluate the association of those aspects of treatment with survival.

## MATERIALS AND METHODS

### Data source

For this analysis, we obtained data from two linked databases. The National Cancer Institute's Surveillance, Epidemiology, and End Results (SEER) cancer registry collects data on cancer patients representing 26% of the US population from five state-based and seven county-based cancer registries. The SEER database includes patient demographics; cancer organ/site, histology, stage and other clinical markers; and primary treatment. The Center for Medicare and Medicaid Services (CMS) maintains a database of patients on Medicare with diagnosis codes and procedural billing codes for their medical expenses. The SEER-Medicare linked database, initiated in 1991, has been previously described and validated (Klabunde *et al*, 2000; Warren *et al*, 2002).

### Patient selection criteria

Eligible patients were those 65 years of age or older at diagnosis with a first primary epithelial OC, American Joint Cancer Committee (AJCC) stage III or IV, and diagnosed between 1 January 1991 and 31 December 2002.

Patients were included in the analysis if they received at least one cycle of chemotherapy within 12 months after surgical resection, survived for at least 7 months after initiating chemotherapy and received chemotherapy continuously for ⩽7 months. Patients who received chemotherapy for >7 months may have had refractory disease, which is not the focus of this study. Patients who did not receive primary, cancer-directed surgery at all or prior to receiving chemotherapy were excluded. Patients who were members of a Medicare health maintenance organization (HMO) at any time from 12 months before to 8 months after their diagnosis of OC also were excluded, because HMOs do not report claims for individual expenses to Medicare. Other exclusion criteria were gaps in Medicare Part A or B coverage during the study period, histologically unconfirmed diagnosis, non-epithelial tumour histology and tumour of low malignant potential. Among 3585 patients who underwent surgery for OC, 1021 did not receive chemotherapy within 12 months of surgery and were excluded.

For each eligible patient, we obtained data on age group at diagnosis, year of diagnosis, race/ethnicity, marital status, tumour stage (III or IV), tumour histology (serous, mucinous, endometrioid, other), comorbidity score, residence (metropolitan or non-metropolitan), type of hospital (teaching or non-teaching) in which they were diagnosed, and socioeconomic indicators for their census tract/zip code of residence from SEER. Comorbidity data and treatment data were obtained from Medicare.

### Treatment delay

The time, in weeks, from surgical resection until delivery of the first cycle of chemotherapy was calculated for each patient. Based on the inclusion criteria for GOG clinical trials, we categorised patients who initiated chemotherapy ⩽6 weeks after surgical resection as receiving timely treatment, and patients who initiated treatment >6 weeks after surgery as receiving delayed treatment. In addition, we categorised patients by 4-week intervals to determine the potential benefit of early initiation of treatment.

### Early termination of treatment

Only women who initiated chemotherapy within 12 weeks after surgery were included in the analysis of early termination of therapy. Treatment duration was defined as the number of days between the first and last claim for chemotherapy. Subjects were categorised as receiving treatment for ⩽3 months or 3–7 months.

### Treatment

We used the International Classification of Diseases, 9th revision, Clinical Modification (ICD-9-CM) codes and AMA Current Procedural Terminology Codes (CPT) to categorise surgical procedures for OC. Among patients who underwent OC-directed surgery, we identified patients who received any cytotoxic chemotherapy in a physician's office or in an outpatient hospital unit.

### Socioeconomic status

An aggregate socioeconomic status (SES) score was generated from a hierarchy of income data from the 2000 Census, according to the method adapted from Nancy Krieger (Bach *et al*, 1999). Patients were ranked on a 1–5 scale, with 1 as the lowest value, based on a formula that incorporated as many of the following variables as were available: median income in the census tract of residence, median income in the zip code of residence, census tract per capita income, zip code per capita income. Patients for whom all values were missing were assigned to the lowest SES category.

### Comorbid disease

To assess the prevalence of comorbid disease in our cohort, we used the Charlson comorbidity index as adapted by Klabunde *et al* (Deyo *et al*, 1992; Klabunde *et al*, 2000, 2002). Medicare inpatient and outpatient claims were searched for ICD-9-CM diagnostic codes that indicated a history of myocardial infarction, congestive heart failure, peripheral vascular disease, cerebrovascular disease, dementia, chronic pulmonary disease, connective tissue disease, mild-to-severe liver disease, diabetes with or without end-organ damage, hemiplegia, moderate-to-severe renal disease, or acquired immunodeficiency syndrome in the Medicare files from 12 months before to 1 month after the diagnosis of cancer. Each condition was weighted, and patients were assigned a score based on the Klabunde–Charlson index (Klabunde *et al*, 2000, 2002).

### Survival

Survival was calculated as the number of months from the cancer diagnosis date to the Medicare date of death. Ovarian-cancer-specific survival was defined as the number of months from the cancer diagnosis to date of death from OC. Patients who survived past the end of follow-up (31 December 2003) were censored and contributed the time from their diagnosis date to the end of follow-up to the analyses of overall survival.

### Statistical analysis

The *χ*^2^ test was used to compare the frequency distributions of categorical variables. All hypothesis tests were two-sided. In a logistic regression analysis, we modelled the predictors of delayed treatment. In Cox proportional hazards analyses, we modelled the overall mortality hazard ratios (HRs) of patients who received delayed treatment compared to other patients, controlling for the other predictor variables. We also generated adjusted Kaplan–Meier curves of overall survival by delayed *vs* timely start of chemotherapy and for termination in ⩽3 *vs* >3 months.

## RESULTS

We identified 6047 individuals with stages III–IV epithelial OC from the SEER-Medicare database. A total of 3585 women underwent OC-directed surgery. Among these women, 2558 (71%) received at least one cycle of chemotherapy. Table 1 shows their demographic characteristics.

### Treatment delay

Among the 2558 patients who received chemotherapy, 1712 (67%) began treatment within 6 weeks of surgery, while 846 (33%) initiated chemotherapy >6 weeks after surgery. The median time to the initiation of chemotherapy was 5 weeks after surgery. In univariate analysis, advanced age, black race, being unmarried and an increased number of comorbid conditions were associated with delayed treatment; living in a metropolitan area, treatment at a teaching hospital, advanced stage, histology, SES and year of diagnosis were not associated with delayed treatment (Table 1). In a logistic regression model, advanced age, black race and marital status were associated with delayed initiation of chemotherapy (Table 2). Black patients were more than twice as likely as white patients to delay initiation of chemotherapy (*P*=0.0007).

In a Cox proportional hazards regression model, treatment delay was associated with a 13% increase in overall mortality (HR=1.13; 95% CI, 1.03–1.25) (Table 2). Advanced age at diagnosis, stage IV disease, mucinous histology and increasing medical comorbidities were also associated with an increased risk of death. Treatment at a teaching hospital was associated with a lower mortality.

**Figure 1:**
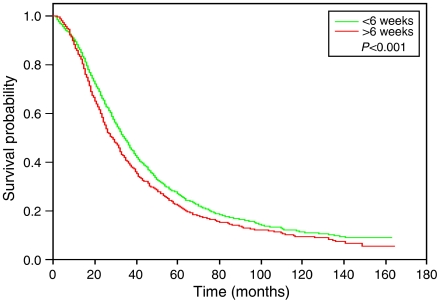
Kaplan–Meier curve for duration interval from surgery to initiation of therapy.

The median survival for women who initiated treatment within 6 weeks was 34 months compared to 28 months for patients who began therapy more than 6 weeks after surgery. Kaplan–Meier analysis demonstrated that women who initiated treatment >6 weeks after had poorer overall (*P*<0.0001) and OC-specific (*P*=0.009) survival than women who did not delay treatment (Figure 1). However, initiating therapy <4 weeks postoperatively provided no survival benefit from doing so at 4–8 weeks (*P*=0.27).

### Early termination of treatment

Among the 1932 patients who initiated therapy within 12 weeks after surgery (Table 3), the median duration of therapy was 3.5 months or ∼15 weeks, consistent with our assumption that if standard treatment with six cycles were administered every 3 weeks, it would take about 15 weeks from initiation to completion of therapy. A total of 714 patients (37%) were treated for ⩽3 months and 1218 (63%) for 3–7 months. In univariate analysis, race, type of hospital and number of medical comorbidities were associated with duration of treatment (Table 3). In a logistic regression model treatment at a teaching hospital was associated with receiving 3–7 months of therapy (Table 4).

**Figure 2:**
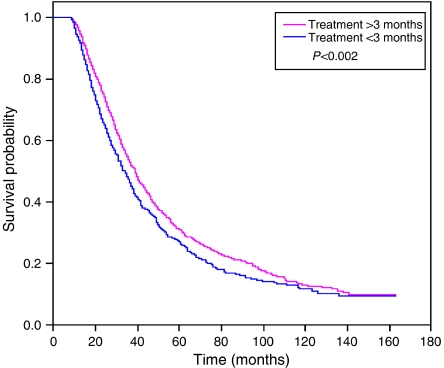
Kaplan–Meier curve for duration chemotherapy.

In a Cox proportional hazards regression model, patients treated for 3–7 months had 16% lower mortality than patients treated for ⩽3 months (HR=0.84; 95% CI, 0.75–0.94) (Table 4). The median survival was 38 months for those treated for 3–7 months compared to 33 months for women treated for <3 months. Figure 2 displays the Kaplan–Meier plots of survival as a function of treatment duration. Treatment for ⩽3 months was associated with poorer overall (*P*<0.0001) and cancer-specific (*P*<0.0001) survival than longer treatment.

## DISCUSSION

Our findings demonstrate that among women with advanced OC who receive chemotherapy, substantial variability exists in the delivery of this treatment. Women who initiated therapy more than 6 weeks after surgery and patients who received an abbreviated course of treatment for less than 3 months were more likely to die even after adjustment for other known prognostic factors. Although waiting longer than 6 weeks to initiate therapy was associated with poorer survival, starting within 4 weeks after surgery was not associated with better survival.

The timing of initiation of adjuvant chemotherapy and survival has been studied in a variety of settings, with varied results. Among patients with lymph node-positive, oestrogen receptor-negative breast tumours, initiation of chemotherapy within 4 weeks was associated with improved survival (Colleoni *et al*, 2000). Elderly patients with early-stage breast cancer appeared to derive no benefit from early initiation of chemotherapy, but long treatment initiation delays were associated with increased mortality (Hershman *et al*, 2006b). Other adjuvant breast cancer trials have also failed to detect a benefit from early initiation of therapy (Shannon *et al*, 2003; Cold *et al*, 2005). Our group recently reported the effect of treatment delay in patients with stage III colon cancer. In a multivariate analysis, delaying initiation of treatment for over 3 months was associated with a 50% increase in colon cancer-specific mortality (Hershman *et al*, 2006a).

A theoretical basis for early administration of cytotoxic agents has been demonstrated in several preclinical models. In a mouse mammary tumour model, removal of the primary lesions resulted in increased tumour proliferation and, therefore, more susceptibility to cytotoxic chemotherapeutic agents. In these studies, early perioperative initiation of chemotherapy resulted in better tumour control than delayed therapy (Fisher *et al*, 1983; Bell *et al*, 1988).

A prospective GOG trial of adjuvant cisplatin and cyclophosphamide with or without doxorubicin examined the effect of delay in the initiation of treatment and outcome for 349 patients with stage III OC. The investigators categorised patients by week of initiation and found that women who began therapy at week 6 had worse survival than those treated earlier (Omura *et al*, 1989). In a multicentre retrospective Italian report as well as a single institution study from the United States, timing to initiation of treatment had no effect on survival (Gadducci *et al*, 2005; Aletti *et al*, 2007). In a separate study of 472 patients, Flynn *et al* (2002) found that women who started chemotherapy within 3 weeks after surgery had a shorter progression-free interval than women who started later. However, the early treatment group had a significantly higher percentage of patients with large volume residual tumours.

Our study differs from previous reports in several respects. We compared patients who initiated treatment 6 weeks or earlier after surgery with women who began treatment later. The majority of previous studies divided patients by much shorter time intervals. Patients in the GOG trial were all treated within 6 weeks and stratified by weekly intervals (Omura *et al*, 1989). Similarly, the majority of patients in the other reports were treated relatively early (Flynn *et al*, 2002; Gadducci *et al*, 2005; Aletti *et al*, 2007). Aletti *et al* (2007) and the Multicenter Italian Study Group divided patients into quartiles, the last quartile consisting of patients treated >34 days after surgery (Omura *et al*, 1989; Gadducci *et al*, 2005). These data suggest that very early treatment may be of limited clinical benefit but that prolonged delays in treatment initiation may have a deleterious effect on survival. Half of our elderly subjects were treated 5 weeks or more after surgery. The relative contributions of patient and physician preferences, performance status and duration of recovery from surgery to delay are unknown.

We found that women treated with chemotherapy for a total duration of <3 months had a 16% higher risk of dying than women treated longer. Similar findings have been reported in both adjuvant colon cancer and breast cancer treatment (Tingulstad *et al*, 2003; Hershman *et al*, 2005). Previous reports have found that patients treated at teaching hospitals were more likely than those at non-teaching hospitals to receive a full six cycles of therapy (Tingulstad *et al*, 2003; Paulsen *et al*, 2006b). In a patterns of care study, Paulsen *et al* (2006b) demonstrated that 43% of patients at non-teaching hospitals but only 16% of patients at teaching hospitals received <6 courses of therapy. Notably, only 72% of women at non-teaching hospitals received some form of chemotherapy. In our cohort, 32% of patients at non-teaching hospitals compared to 38% at teaching hospitals received <3 months of therapy. While statistically significant, the difference was quite small.

This study is one of the first to examine age and racial differences in the quality of chemotherapy delivery in women with OC. Advancing age was associated with a progressively greater risk of delay in initiation of chemotherapy. Previous work has suggested that elderly OC patients are less likely to receive chemotherapy than younger patients and that those who do are often treated with less-aggressive regimens (Bruchim *et al*, 2002; Efstathiou *et al*, 2007). A recent study of breast cancer patients suggested that black patients and elderly patients were more likely to receive nonstandard treatment regimen (Griggs *et al*, 2007). Similarly, in our series, black patients were more likely to experience a delay in starting chemotherapy. Previous univariate analysis of SEER data suggested that black patients were less likely to receive guideline-based chemotherapy (Harlan *et al*, 2003).

Treatment quality is influenced by physician-related factors. Several studies have demonstrated superior outcomes for women treated by surgical specialists and high-volume surgeons (Junor *et al*, 1999; Carney *et al*, 2002; Elit *et al*, 2002; Earle *et al*, 2006; Goff *et al*, 2006, 2007; Paulsen *et al*, 2006b; Vernooij *et al*, 2007). In an analysis of over 3000 patients, those operated on by gynaecologic oncologists were more likely to undergo surgical cytoreduction and had improved outcomes (Earle *et al*, 2006). Likewise, patients who were operated on at high-volume facilities and teaching hospitals are more likely to undergo comprehensive surgical staging and cytoreduction, and have better survival than those treated at smaller and non-teaching hospitals (Tingulstad *et al*, 2003; Paulsen *et al*, 2006b; Vernooij *et al*, 2007). However, many patients with OC do not receive care from surgical specialists or at large volume facilities (Carney *et al*, 2002; Bristow *et al*, 2004).

Although the patients in the SEER-Medicare database are representative of elderly patients treated in the United States, the database has a number of limitations. It does not include patients under the age of 65 years. It does not include data on important prognostic factors, such as the volume of residual tumour at the initiation of chemotherapy; the actual doses delivered (we used duration of therapy as a surrogate for dose intensity); patient and physician preferences, which are critically important to both the initiation and continuation of chemotherapy; and performance status. Although we used age and medical comorbidities as surrogates, these variables are unable to compensate fully for the lack of performance status information.

Our findings indicate that delayed treatment initiation and early chemotherapy discontinuation were common in elderly women with OC, and that these variations in treatment delivery are associated with survival. Although early initiation of chemotherapy appears to confer little benefit, prolonged delays in beginning treatment and early discontinuation of treatment are associated with poor outcomes. Prospective studies of factors that influence the quality of care in women with OC are needed, but until such studies are completed, efforts should be made to facilitate prompt initiation and full completion of adjuvant chemotherapy.

## Figures and Tables

**Table 1:** Univariate associations between weeks to initiation of chemotherapy and demographic and clinical variables

	**⩽6**		**6**		**Total**		
**Months to chemotherapy**	***N***	**%**	***N***	**%**	***N***	**%**	***P*-value**
	1712	(67)	846	(33)	2558	(100)	
*Age at diagnosis*							
65–69	475	(28)	203	(24)	678	(27)	
70–74	593	(35)	259	(31)	852	(33)	
75–79	429	(25)	242	(29)	671	(26)	
80+	215	(13)	142	(17)	357	(14)	0.0002
							
*Race*							
White	1598	(94)	756	(89)	2354	(92)	
Black	42	(2)	44	(5)	86	(3)	
Other	72	(4)	46	(5)	118	(5)	0.0004
							
*Live in metropolitan location*							
No	159	(9)	67	(8)	226	(9)	
Yes	1553	(91)	779	(92)	2332	(91)	0.25
							
*Marital status*							
No	752	(44)	426	(50)	1178	(46)	
Yes	916	(54)	395	(47)	1311	(51)	
Unknown	44	(3)	25	(3)	69	(3)	0.005
							
*Teaching hospital*							
No	302	(18)	166	(20)	468	(18)	
Yes	1410	(82)	680	(80)	2090	(82)	0.22
							
*Histology*							
Serous	1066	(62)	508	(60)	1574	(62)	
Mucinous	58	(3)	40	(5)	98	(4)	
Endometrioid	150	(9)	59	(7)	209	(8)	
Other	438	(26)	239	(28)	677	(26)	0.077
							
*Stage*							
III	1065	(62)	530	(63)	1595	(62)	
IV	647	(38)	316	(37)	963	(38)	0.83
							
*No. of comorbid conditions*							
0	894	(52)	403	(48)	1297	(51)	
1	475	(28)	241	(28)	716	(28)	
⩾2	343	(20)	202	(24)	545	(21)	0.03
							
*SES*							
Lowest quintile	318	(19)	156	(18)	474	(19)	
Second quintile	324	(19)	161	(19)	485	(19)	
Third quintile	343	(20)	172	(20)	515	(20)	
Fourth quintile	338	(20)	174	(21)	512	(20)	
Fifth quintile	389	(23)	183	(22)	572	(22)	0.97

**Table 2:** Association of demographic and clinical characteristics with initiation of chemotherapy >6 weeks after surgery and overall mortality hazard ratios in patients with ovarian cancer by time to initiation of chemotherapy

	**>6 weeks to initiation of treatment**	**Overall mortality**
	Odds ratio* (95% CI)	Hazard ratio (95% CI)*
*Initiation of treatment*		
⩽6 weeks	—	Referent
>6 weeks	—	1.13 (1.03–1.25)^*^
		
*Age at diagnosis*		
65–70	Referent	Referent
70–74	1.02 (0.81–1.27)	1.23 (1.09–1.38)^*^
75–79	1.31 (1.04–1.65)^*^	1.27 (1.12–1.44)^*^
80+	1.47 (1.11–1.93)	1.45 (1.24–1.68)^*^
		
*Race*		
White	Referent	Referent
Black	2.24 (1.42–3.52)^*^	0.80 (0.62–1.05)
Other	1.38 (0.94–2.03)	0.82 (0.66–1.03)
		
*Stage*		
III	Referent	Referent
IV	0.95 (0.80–1.1)	1.22 (1.12–1.34)^*^
		
*Histology*		
Serous	Referent	Referent
Mucinous	1.37 (0.89–2.09)	1.42 (1.13–1.78)^*^
Endometrioid	0.80 (0.58–1.11)	0.91 (0.77–1.08)
Other	1.17 (0.96–1.42)	1.08 (0.97–1.20)
		
*Live in a metropolitan area*		
Yes	Referent	Referent
No	0.89 (0.65–1.24)	1.1 (0.9–1.3)
		
*No. of comorbid conditions*		
0	Referent	Referent
1	1.13 (0.92–1.37)	1.08 (0.97–1.20)
⩾2	1.23 (0.99–1.52)^*^	1.31 (1.17–1.47)^*^
		
*SES*		
Lowest quintile	Referent	Referent
Second quintile	1.12 (0.85–1.48)	1.01 (0.87–1.18)
Third quintile	1.13 (0.85–1.49)	1.03 (0.89–1.20)
Fourth quintile	1.19 (0.89–1.58)	0.93 (0.79–1.08)
Fifth quintile	1.09 (0.82–1.44)	0.91 (0.79–1.06)
		
*Marital status*		
Yes	Referent	Referent
No	1.19 (1.00–1.42)^*^	1.03 (0.94–1.13)
Unknown	1.31 (0.78–2.19)	0.96 (0.72–1.28)
		
*Teaching hospital*		
No	Referent	Referent
Yes	0.88 (0.71–1.09)	0.82 (0.73–0.92)^*^

All variables controlled for each other, and year of diagnosis.

^*^P<0.01.

**Table 3:** Univariate association between length of chemotherapy treatment (months) and demographic and clinical variables

	**⩽3 months**		**3–7 months**		**Total**		
**Months of chemotherapy**	***N***	**%**	***N***	**%**	***N***	**%**	***P*-value**
	714	(37)	1218	(63)	1932	(100)	
*Age at diagnosis*							
65–69	202	(28)	325	(27)	527	(27)	
70–74	218	(31)	428	(35)	646	(33)	
75–79	190	(27)	309	(25)	499	(26)	
80+	104	(15)	156	(13)	260	(13)	0.20
							
*Race*							
White	648	(91)	1141	(94)	1789	(93)	
Black	29	(4)	26	(2)	55	(3)	
Other	37	(5)	51	(4)	88	(5)	0.03
							
*Live in metropolitan location*							
No	50	(7)	112	(9)	162	(8)	
Yes	664	(93)	1106	(91)	1770	(92)	0.09
							
*Marital status*							
No	341	(48)	531	(44)	872	(45)	
Yes	350	(49)	652	(54)	1002	(52)	
Unknown	23	(3)	35	(3)	58	(3)	0.16
							
*Teaching hospital*							
No	106	(15)	229	(19)	335	(17)	
Yes	608	(85)	989	(81)	1597	(83)	0.03
							
*Histology*							
Serous	449	(63)	776	(64)	1225	(63)	
Mucinous	13	(2)	35	(3)	48	(2)	
Endometrioid	60	(8)	97	(8)	157	(8)	
Other	192	(27)	310	(25)	502	(26)	0.47
							
*Stage*							
III	447	(63)	775	(64)	1222	(63)	
IV	267	(37)	443	(36)	710	(37)	0.65
							
*No. of comorbid conditions*							
0	357	(50)	646	(53)	1003	(52)	
1	198	(28)	362	(30)	560	(29)	
⩾2	159	(22)	210	(17)	369	(19)	0.02
							
*SES*							
Lowest quintile	131	(18)	208	(17)	339	(18)	
Second quintile	137	(19)	231	(19)	368	(19)	
Third quintile	154	(22)	239	(20)	393	(20)	
Fourth quintile	153	(21)	246	(20)	399	(21)	
Fifth quintile	139	(19)	294	(24)	433	(22)	0.20

**Table 4:** Association of demographic and clinical characteristics with duration of chemotherapy ⩽3 months and mortality hazard ratios in patients with ovarian cancer by duration of chemotherapy

	**⩽3 months treatment**	**Overall mortality**
	Odds ratio* (95% CI)	Hazard ratio (95% CI)*
*Length of treatment*		
<3 months	—	Referent
3–7 months	—	0.84 (0.75–0.94)^*^
		
*Age at diagnosis*		
65–70	Referent	Referent
70–74	1.23 (0.96–1.57)	1.29 (1.12–1.48)^*^
75–79	1.02 (0.79–1.32)	1.29 (1.11–1.49)^*^
80+	0.96 (0.70–1.32)	1.49 (1.25–1.79)^*^
		
*Race*		
White	Referent	Referent
Black	0.60 (0.34–1.05)	0.83 (0.59–1.17)
Hispanic	2.30 (0.72–7.28)	1.24 (0.65–2.36)
Other	0.69 (0.43–1.13)	0.89 (0.66–1.20)
		
*Stage*		
III	Referent	Referent
IV	0.97 (0.80–1.18)	1.26 (1.13–1.40)^*^
		
*Histology*		
Serous	Referent	Referent
Mucinous	1.71 (0.89–3.32)	0.95 (0.67–1.44)
Endometrioid	0.95 (0.67–1.35)	0.80 (0.65–0.98)
Other	0.92 (0.74–1.14)	1.01 (0.89–1.15)
		
*Live in a metropolitan area*		
Yes	Referent	Referent
No	1.40 (0.96–2.04)	1.07 (0.86–1.32)
		
*No. of comorbid conditions*		
0	Referent	Referent
1	1.05 (0.84–1.30)	1.12 (0.99–1.27)
⩾2	0.78 (0.61–1.00)	1.19 (1.03–1.36)^*^
		
*SES*		
Lowest quintile	Referent	Referent
Second quintile	1.08 (0.79–1.47)	1.04 (0.87–1.24)
Third quintile	1.02 (0.74–1.40)	1.17 (0.98–1.40)
Fourth quintile	1.05 (0.76–1.44)	0.94 (0.78–1.13)
Fifth quintile	1.36 (0.99–1.87)	0.93 (0.77–1.11)
		
*Marital status*		
Yes	Referent	Referent
No	0.88 (0.73–1.07)	1.10 (0.98–1.22)
Unknown	0.83 (0.48–1.43)	1.12 (0.81–1.55)
		
*Teaching hospital*		
No	Referent	Referent
Yes	0.75 (0.58–0.97)	0.83 (0.73–0.96)^*^

All variables controlled for each other and year of diagnosis.

^*^P<0.01.

## References

[bib1] Alberts DS, Liu PY, Hannigan EV, O’Toole R, Williams SD, Young JA, Franklin EW, Clarke-Pearson DL, Malviya VK, DuBeshter B (1996) Intraperitoneal cisplatin plus intravenous cyclophosphamide *vs* intravenous cisplatin plus intravenous cyclophosphamide for stage III ovarian cancer. N Engl J Med 335: 1950–1955 8960474 10.1056/NEJM199612263352603

[bib2] Aletti GD, Long HJ, Podratz KC, Cliby WA (2007) Is time to chemotherapy a determinant of prognosis in advanced-stage ovarian cancer? Gynecol Oncol 104: 212–216 17023033 10.1016/j.ygyno.2006.07.045

[bib3] Armstrong DK, Bundy B, Wenzel L, Huang HQ, Baergen R, Lele S, Copeland LJ, Walker JL, Burger RA (2006) Intraperitoneal cisplatin and paclitaxel in ovarian cancer. N Engl J Med 354: 34–43 16394300 10.1056/NEJMoa052985

[bib4] Bach PB, Cramer LD, Warren JL, Begg CB (1999) Racial differences in the treatment of early-stage lung cancer. N Engl J Med 341: 1198–1205 10519898 10.1056/NEJM199910143411606

[bib5] Bell RS, Roth YF, Gebhardt MC, Bell DF, Rosenberg AE, Mankin HJ, Suit HD (1988) Timing of chemotherapy and surgery in a murine osteosarcoma model. Cancer Res 48: 5533–5538 3166399

[bib6] Bristow RE, Zahurak ML, del Carmen MG, Gordon TA, Fox HE, Trimble EL, Montz FJ (2004) Ovarian cancer surgery in Maryland: volume-based access to care. Gynecol Oncol 93: 353–360 15099945 10.1016/j.ygyno.2004.02.010

[bib7] Bruchim I, Altaras M, Fishman A (2002) Age contrasts in clinical characteristics and pattern of care in patients with epithelial ovarian cancer. Gynecol Oncol 86: 274–278 12217748 10.1006/gyno.2002.6759

[bib8] Carney ME, Lancaster JM, Ford C, Tsodikov A, Wiggins CL (2002) A population-based study of patterns of care for ovarian cancer: who is seen by a gynecologic oncologist and who is not? Gynecol Oncol 84: 36–42 11748973 10.1006/gyno.2001.6460

[bib9] Citron ML, Berry DA, Cirrincione C, Hudis C, Winer EP, Gradishar WJ, Davidson NE, Martino S, Livingston R, Ingle JN, Perez EA, Carpenter J, Hurd D, Holland JF, Smith BL, Sartor CI, Leung EH, Abrams J, Schilsky RL, Muss HB, Norton L (2003) Randomized trial of dose-dense *vs* conventionally scheduled and sequential *vs* concurrent combination chemotherapy as postoperative adjuvant treatment of node-positive primary breast cancer: first report of Intergroup Trial C9741/Cancer and Leukemia Group B Trial 9741. J Clin Oncol 21: 1431–1439 12668651 10.1200/JCO.2003.09.081

[bib10] Cold S, During M, Ewertz M, Knoop A, Moller S (2005) Does timing of adjuvant chemotherapy influence the prognosis after early breast cancer? Results of the Danish Breast Cancer Cooperative Group (DBCG). Br J Cancer 93: 627–632 16136052 10.1038/sj.bjc.6602734 PMC2361615

[bib11] Colleoni M, Bonetti M, Coates AS, Castiglione-Gertsch M, Gelber RD, Price K, Rudenstam CM, Lindtner J, Collins J, Thurlimann B, Holmberg S, Veronesi A, Marini G, Goldhirsch A (2000) Early start of adjuvant chemotherapy may improve treatment outcome for premenopausal breast cancer patients with tumors not expressing estrogen receptors. The International Breast Cancer Study Group. J Clin Oncol 18: 584–590 10653873 10.1200/JCO.2000.18.3.584

[bib12] Conte PF, Bruzzone M, Carnino F, Gadducci A, Algeri R, Bellini A, Boccardo F, Brunetti I, Catsafados E, Chiara S, Foglia G, Gallo L, Iskra L, Mammoliti S, Parodi G, Ragni N, Rosso R, Rugiati S, Rubagotti A (1996) High-dose *vs* low-dose cisplatin in combination with cyclophosphamide and epidoxorubicin in suboptimal ovarian cancer: a randomized study of the Gruppo Oncologico Nord-Ovest. J Clin Oncol 14: 351–356 8636743 10.1200/JCO.1996.14.2.351

[bib13] Deyo RA, Cherkin DC, Ciol MA (1992) Adapting a clinical comorbidity index for use with ICD-9-CM administrative databases. J Clin Epidemiol 45: 613–619 1607900 10.1016/0895-4356(92)90133-8

[bib14] Earle CC, Schrag D, Neville BA, Yabroff KR, Topor M, Fahey A, Trimble EL, Bodurka DC, Bristow RE, Carney M, Warren JL (2006) Effect of surgeon specialty on processes of care and outcomes for ovarian cancer patients. J Natl Cancer Inst 98: 172–180 16449677 10.1093/jnci/djj019

[bib15] Efstathiou E, Dimopoulos MA, Bozas G, Kastritis E, Moulopoulos LA, Rodolakis A, Vlahos G, Gika D, Papadimitriou C, Bamias A (2007) Advanced epithelial ovarian cancer in the elderly: chemotherapy tolerance and outcome. Anticancer Res 27: 611–617 17348450

[bib16] Elit L, Bondy SJ, Paszat L, Przybysz R, Levine M (2002) Outcomes in surgery for ovarian cancer. Gynecol Oncol 87: 260–267 12468323 10.1006/gyno.2002.6834

[bib17] Fisher B, Gunduz N, Saffer EA (1983) Influence of the interval between primary tumor removal and chemotherapy on kinetics and growth of metastases. Cancer Res 43: 1488–1492 6831397

[bib18] Flynn PM, Paul J, Cruickshank DJ (2002) Does the interval from primary surgery to chemotherapy influence progression-free survival in ovarian cancer? Gynecol Oncol 86: 354–357 12217760 10.1006/gyno.2002.6750

[bib19] Gadducci A, Sartori E, Landoni F, Zola P, Maggino T, Maggioni A, Cosio S, Frassi E, LaPresa MT, Fuso L, Cristofani R (2005) Relationship between time interval from primary surgery to the start of taxane- plus platinum-based chemotherapy and clinical outcome of patients with advanced epithelial ovarian cancer: results of a multicenter retrospective Italian study. J Clin Oncol 23: 751–758 15613698 10.1200/JCO.2005.03.065

[bib20] Goff BA, Matthews BJ, Larson EH, Andrilla CH, Wynn M, Lishner DM, Baldwin LM (2007) Predictors of comprehensive surgical treatment in patients with ovarian cancer. Cancer 109: 2031–2042 17420977 10.1002/cncr.22604

[bib21] Goff BA, Matthews BJ, Wynn M, Muntz HG, Lishner DM, Baldwin LM (2006) Ovarian cancer: patterns of surgical care across the United States. Gynecol Oncol 103: 383–390 17005244 10.1016/j.ygyno.2006.08.010

[bib22] Gore M, Mainwaring P, A’Hern R, MacFarlane V, Slevin M, Harper P, Osborne R, Mansi J, Blake P, Wiltshaw E, Shepherd J (1998) Randomized trial of dose-intensity with single-agent carboplatin in patients with epithelial ovarian cancer. London Gynaecological Oncology Group. J Clin Oncol 16: 2426–2434 9667260 10.1200/JCO.1998.16.7.2426

[bib23] Griggs JJ, Culakova E, Sorbero ME, Poniewierski MS, Wolff DA, Crawford J, Dale DC, Lyman GH (2007) Social and racial differences in selection of breast cancer adjuvant chemotherapy regimens. J Clin Oncol 25: 2522–2527 17577029 10.1200/JCO.2006.10.2749

[bib24] Harlan LC, Clegg LX, Trimble EL (2003) Trends in surgery and chemotherapy for women diagnosed with ovarian cancer in the United States. J Clin Oncol 21: 3488–3494 12972525 10.1200/JCO.2003.01.061

[bib25] Hershman D, Hall MJ, Wang X, Jacobson JS, McBride R, Grann VR, Neugut AI (2006a) Timing of adjuvant chemotherapy initiation after surgery for stage III colon cancer. Cancer 107: 2581–2588 17078055 10.1002/cncr.22316

[bib26] Hershman D, McBride R, Jacobson JS, Lamerato L, Roberts K, Grann VR, Neugut AI (2005) Racial disparities in treatment and survival among women with early-stage breast cancer. J Clin Oncol 23: 6639–6646 16170171 10.1200/JCO.2005.12.633

[bib27] Hershman DL, Wang X, McBride R, Jacobson JS, Grann VR, Neugut AI (2006b) Delay of adjuvant chemotherapy initiation following breast cancer surgery among elderly women. Breast Cancer Res Treat 99: 313–321 16583264 10.1007/s10549-006-9206-z

[bib28] Hudis C, Fornier M, Riccio L, Lebwohl D, Crown J, Gilewski T, Surbone A, Currie V, Seidman A, Reichman B, Moynahan M, Raptis G, Sklarin N, Theodoulou M, Weiselberg L, Salvaggio R, Panageas KS, Yao TJ, Norton L (1999) 5-year results of dose-intensive sequential adjuvant chemotherapy for women with high-risk node-positive breast cancer: a phase II study. J Clin Oncol 17: 1118 10561169 10.1200/JCO.1999.17.4.1118

[bib29] Jakobsen A, Bertelsen K, Andersen JE, Havsteen H, Jakobsen P, Moeller KA, Nielsen K, Sandberg E, Stroeyer I (1997) Dose-effect study of carboplatin in ovarian cancer: a Danish Ovarian Cancer Group study. J Clin Oncol 15: 193–198 8996142 10.1200/JCO.1997.15.1.193

[bib30] Junor EJ, Hole DJ, McNulty L, Mason M, Young J (1999) Specialist gynaecologists and survival outcome in ovarian cancer: a Scottish national study of 1866 patients. Br J Obstet Gynaecol 106: 1130–1136 10549956 10.1111/j.1471-0528.1999.tb08137.x

[bib31] Klabunde CN, Potosky AL, Legler JM, Warren JL (2000) Development of a comorbidity index using physician claims data. J Clin Epidemiol 53: 1258–1267 11146273 10.1016/s0895-4356(00)00256-0

[bib32] Klabunde CN, Warren JL, Legler JM (2002) Assessing comorbidity using claims data: an overview. Med Care 40, IV- 26–35 12187165 10.1097/00005650-200208001-00004

[bib33] Markman M, Bundy BN, Alberts DS, Fowler JM, Clark-Pearson DL, Carson LF, Wadler S, Sickel J (2001) Phase III trial of standard-dose intravenous cisplatin plus paclitaxel *vs* moderately high-dose carboplatin followed by intravenous paclitaxel and intraperitoneal cisplatin in small-volume stage III ovarian carcinoma: an intergroup study of the Gynecologic Oncology Group, Southwestern Oncology Group, and Eastern Cooperative Oncology Group. J Clin Oncol 19: 1001–1007 11181662 10.1200/JCO.2001.19.4.1001

[bib34] McGuire WP, Hoskins WJ, Brady MF, Homesley HD, Creasman WT, Berman ML, Ball H, Berek JS, Woodward J (1995) Assessment of dose-intensive therapy in suboptimally debulked ovarian cancer: a Gynecologic Oncology Group study. J Clin Oncol 13: 1589–1599 7602348 10.1200/JCO.1995.13.7.1589

[bib35] McGuire WP, Hoskins WJ, Brady MF, Kucera PR, Partridge EE, Look KY, Clarke-Pearson DL, Davidson M (1996) Cyclophosphamide and cisplatin compared with paclitaxel and cisplatin in patients with stage III and stage IV ovarian cancer. N Engl J Med 334: 1–6 7494563 10.1056/NEJM199601043340101

[bib36] McGuire WP (1997) How many more nails to seal the coffin of dose intensity? Ann Oncol 8: 311–313 9209658 10.1023/a:1008211203563

[bib37] Omura GA, Bundy BN, Berek JS, Curry S, Delgado G, Mortel R (1989) Randomized trial of cyclophosphamide plus cisplatin with or without doxorubicin in ovarian carcinoma: a Gynecologic Oncology Group Study. J Clin Oncol 7: 457–465 2926470 10.1200/JCO.1989.7.4.457

[bib38] Paulsen T, Kaern J, Kjaerheim K, Haldorsen T, Trope C (2006a) Influence of interval between primary surgery and chemotherapy on short-term survival of patients with advanced ovarian, tubal or peritoneal cancer. Gynecol Oncol 102: 447–452 16516277 10.1016/j.ygyno.2006.01.035

[bib39] Paulsen T, Kjaerheim K, Kaern J, Tretli S, Trope C (2006b) Improved short-term survival for advanced ovarian, tubal, and peritoneal cancer patients operated at teaching hospitals. Int J Gynecol Cancer 16(Suppl 1): 11–17 16515561 10.1111/j.1525-1438.2006.00319.x

[bib40] Schrag D, Earle C, Xu F, Panageas KS, Yabroff KR, Bristow RE, Trimble EL, Warren JL (2006) Associations between hospital and surgeon procedure volumes and patient outcomes after ovarian cancer resection. J Natl Cancer Inst 98: 163–171 16449676 10.1093/jnci/djj018

[bib41] Shannon C, Ashley S, Smith IE (2003) Does timing of adjuvant chemotherapy for early breast cancer influence survival? J Clin Oncol 21: 3792–3797 14551298 10.1200/JCO.2003.01.073

[bib42] Sundararajan V, Hershman D, Grann VR, Jacobson JS, Neugut AI (2002) Variations in the use of chemotherapy for elderly patients with advanced ovarian cancer: a population-based study. J Clin Oncol 20: 173–178 11773167 10.1200/JCO.2002.20.1.173

[bib43] Tingulstad S, Skjeldestad FE, Hagen B (2003) The effect of centralization of primary surgery on survival in ovarian cancer patients. Obstet Gynecol 102: 499–505 12962932 10.1016/s0029-7844(03)00579-9

[bib44] Vernooij F, Heintz P, Witteveen E, van der Graaf Y (2007) The outcomes of ovarian cancer treatment are better when provided by gynecologic oncologists and in specialized hospitals: a systematic review. Gynecol Oncol 105: 801–812 17433422 10.1016/j.ygyno.2007.02.030

[bib45] Warren JL, Harlan LC, Fahey A, Virnig BA, Freeman JL, Klabunde CN, Cooper GS, Knopf KB (2002) Utility of the SEER-Medicare data to identify chemotherapy use. Med Care 40, IV- 55–61 10.1097/01.MLR.0000020944.17670.D7 12187169

[bib46] Warwick J, Kehoe S, Earl H, Luesley D, Redman C, Chan KK (1995) Long-term follow-up of patients with advanced ovarian cancer treated in randomised clinical trials. Br J Cancer 72: 1513–1517 8519669 10.1038/bjc.1995.539 PMC2034090

